# Enhanced Copper Bonding Interfaces by Quenching to Form Wrinkled Surfaces

**DOI:** 10.3390/nano14100861

**Published:** 2024-05-15

**Authors:** Tsan-Feng Lu, Yu-Ting Yen, Pei-Wen Wang, Yuan-Fu Cheng, Cheng-Hsiang Chen, YewChung Sermon Wu

**Affiliations:** Department of Materials Science and Engineering, National Yang Ming Chiao Tung University, Hsinchu 30010, Taiwan; s0881513.c@nycu.edu.tw (T.-F.L.); yu.en11@nycu.edu.tw (Y.-T.Y.); nycu3116.en11@nycu.edu.tw (P.-W.W.); weddie6231.11@nycu.edu.tw (Y.-F.C.); rick.chen.en11@nycu.edu.tw (C.-H.C.)

**Keywords:** Cu–Cu direct bonding, wrinkled surface, grain growth, quenching treatment, strain energy

## Abstract

For decades, Moore’s Law has been approaching its limits, posing a huge challenge for further downsizing to nanometer dimensions. A promising avenue to replace Moore’s Law lies in three-dimensional integrated circuits, where Cu–Cu bonding plays a critical role. However, the atomic diffusion rate is notably low at temperatures below 300 °C, resulting in a distinct weak bonding interface, which leads to reliability issues. In this study, a quenching treatment of the Cu film surface was investigated. During the quenching treatment, strain energy was induced due to the variation in thermal expansion coefficients between the Si substrate and the Cu film, resulting in a wrinkled surface morphology on the Cu film. Grain growth was observed at the Cu–Cu bonding interface following bonding at 300 °C for 2 and 4 h. Remarkably, these procedures effectively eliminated the bonding interface.

## 1. Introduction

Moore’s Law has fueled remarkable growth in transistor integration, driving significant technological and economic progress. This law, which dictates the doubling of transistor counts on integrated chips every 24 months [1], has been validated across a wide range of high-performance computing systems, including CPUs, GPUs, FPGAs, mobile application processors, and specialized artificial intelligence (AI) accelerators. However, further dimensional scaling has become increasingly challenging, leading to diminishing returns in transistor costs, power, and performance.

For decades, Moore’s Law has approached its limits, posing significant challenges to further scaling down. In order to comply with Moore’s Law and enhance transistor performance, the concept of the vertical integration of a three-dimensional integrated circuit (3D IC) was proposed [2,3,4,5]. By vertically integrating IC layers within the same package, 3D IC technology offered several advantages, including shorter interconnection wire lengths, a smaller form factor, lower power consumption, and the ability for heterogeneous integration.

Solders have been a standard choice for interconnects in microelectronic devices for decades [6]. However, meeting the increasingly high demands for input/output (I/O) density and reliability poses a challenge for solder interconnects. To overcome these issues, Cu-to-Cu direct bonding was developed [7,8,9,10]. It is currently regarded as one of the most advanced packaging technologies, offering the capability to shrink interconnect sizes down below submicron, while maintaining superior electrical and thermal conductivity [11,12]. The main mechanism of Cu–Cu bonding involves solid diffusion. Nevertheless, the rate of atomic diffusion is notably low at lower temperatures. If bonding temperatures are below 300 °C, minimal grain growth is observed in Cu joints, preserving the sharp interfaces of the original bonding surfaces. Lim et al. [13] found that eliminating the original bonding interface can increase the shear strength of Cu joints by 77%. Thus, eliminating the bonding interface is crucial for enhancing the electrical conductivity and mechanical strength of Cu joints.

It has been widely recognized that when small strains, which are below the levels required for recrystallization, are applied, they can have significant effects on abnormal grain growth (AGG) of polycrystalline Cu [14]. Additionally, AGG has been widely employed to eliminate the Cu–Cu bonding interface, with the aim of enhancing bonding strength and reliability [15,16]. Therefore, we propose a quenching treatment method to introduce strain energy into Cu films. By increasing the stored strain energy, the bonding interface was eliminated via AGG at 300 °C.

## 2. Experimental

### 2.1. Cu Film Electrodeposition

An electroplated Cu film on Si wafer was used in this study. The Si substrate consisted of a 70 nm SiO_2_ layer, with a 20 nm Ta layer sputtered onto it as the adhesion layer. Following this, a 600 nm thick Cu seed layer was sputtered. Subsequently, electroplating of 1.4 μm Cu film was carried out. The surface of the Cu film was then flattened using a chemical mechanical polishing (CMP) process.

### 2.2. Sample Pretreatment

Before the bonding process, wafers were diced into 1 × 1 cm^2^ pieces. Two types of Cu films were employed to investigate the impact of the quenching process on Cu bonding: Cu film without quenching (referred to as BCu) and quenched Cu film (referred to as QBCu).

In the BCu fabrication process, sample pieces underwent a series of cleaning steps. Initially, ultrasonic cleaning with acetone was carried out, followed by treatment with a citric acid solution to eliminate oxides. Subsequently, the samples were rinsed with acetone and deionized (DI) water, and the process concluded with purging using N_2_ gas.

The preparation of QBCu involved several steps. Initially, BCu samples were heated on a hot plate at 250 °C for 5 min, followed by transferring them to an aluminum plate at 0 °C for rapid cooling, with a cooling period lasting 2 min. Subsequently, the samples were immersed in a citric acid solution, rinsed with acetone and DI water, and finally purged using N_2_ gas to conclude the process.

### 2.3. Bonding Process

After the pretreatment, two types of samples were stacked in a differential thermal expansion fixture made of aluminum and stainless steel for the bonding process: (a) B/B (BCu-to-BCu bonding) and (b) QB/B (QBCu-to-BCu bonding). This fixture was identical to the one proposed in our previous work [17]. The bonding temperature and time duration were set at 300 °C for 1, 2, and 4 h, under ordinary vacuum conditions (10^−3^ torr). As the processing temperature increased, the compressive stress on the sample stack also increased due to the differential thermal expansion among the various materials of the fixture. The calculated compressive stress was 65.56 MPa at 300 °C. However, determining the actual stress proved challenging as the Cu films underwent plastic deformation (creep) at elevated temperatures.

### 2.4. Material Characterizations

Before bonding, the surface roughness was measured using atomic force microscopy (AFM, Bruker Dimension Icon Scanning Probe Microscope (ICON), Bruker, Billerica, MA, USA) with a 10 × 10 µm^2^ scan area. The surface morphology of the copper films was confirmed by scanning electron microscope (SEM) and scanning ion microscopy (SIM) images obtained using a dual-beam focused ion beam (FIB, Helios NanoLab 650, FEI, Hillsboro, OR, USA). The Cu crystallographic orientation, grain size, and local strain were investigated using a SEM (JSM-7800F PRIME, Japan Electron Optics Laboratory Co., Ltd., Tokyo, Japan) with an electron backscattered diffraction system (EBSD, Nordlys Max3 EBSD detector, Oxford Instruments, Abingdon-on-Thames, UK) operated at 20 kV. The EBSD data were analyzed using OIM software (TSL, Inc., Draper, UT, USA), which can further analyze local strain and misorientation, such as kernel average misorientation (KAM). The mean grain size was determined from plan-view EBSD of the surface of the Cu films measuring grains in a 15 × 15 µm^2^ EBSD image. To observe the effect of Cu grain growth, the microstructure of the bonding interface was examined using a FIB.

## 3. Results and Discussion

### 3.1. Surface Roughness and Morphology of Cu Films

**Figure 1:**
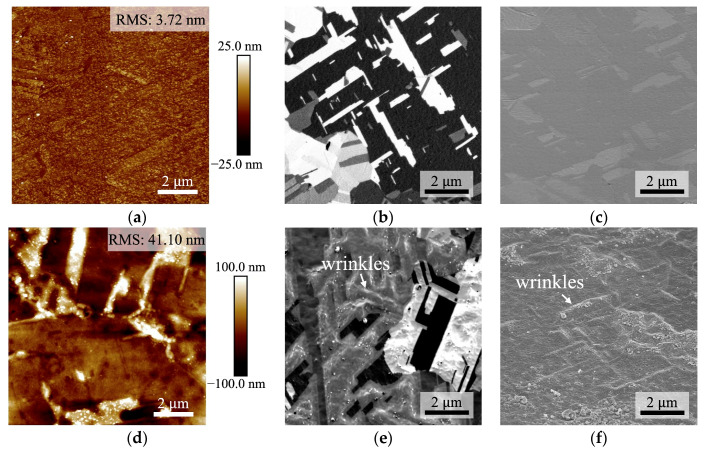
AFM topography images of: (**a**) BCu and (**d**) QBCu. The RMS roughness values were 3.72 and 41.10 nm, respectively. Plane-view SIM images of (**b**) BCu and (**e**) QBCu. SEM images show (**c**) BCu and (**f**) QBCu in the imaging position of 52° tilt. We can observe significant wrinkle morphology in (**e**,**f**).

The surface roughness of the Cu films was measured using AFM. The root mean square (RMS) value for BCu was determined to be 3.72 nm, while for QBCu, it measured 41.10 nm, as shown in Figure 1. Following the quenching treatment, the surface of QBCu exhibited increased roughness, with a notable rise in the RMS value.

The surface morphology was also investigated using a FIB. As shown in Figure 1b,c, the untreated surface, BCu, exhibited a smooth morphology. After the quenching treatment, the surface of QBCu became significantly roughened with a wrinkled surface morphology, as shown in Figure 1e,f.

### 3.2. Effect of Strain Energy on Cu Films

The formation of wrinkles on the surface of QBCu was attributed to the differential thermal expansion between the Si substrate and the Cu film. Upon quenching from 250 °C to 0 °C, the Cu film rapidly contracted, inducing strains along its surface. The large strain energy resulted in the formation of wrinkles, overcoming the activation energy required for wrinkle formation. Contraction stress may distort the local crystal symmetry and introduce bond strain and crystallographic defects in the Cu film. The detailed mechanism of wrinkle formation has been thoroughly discussed in Refs. [18,19,20,21]. In this study, we further validated the induced local strain by employing KAM analysis.

**Figure 2:**
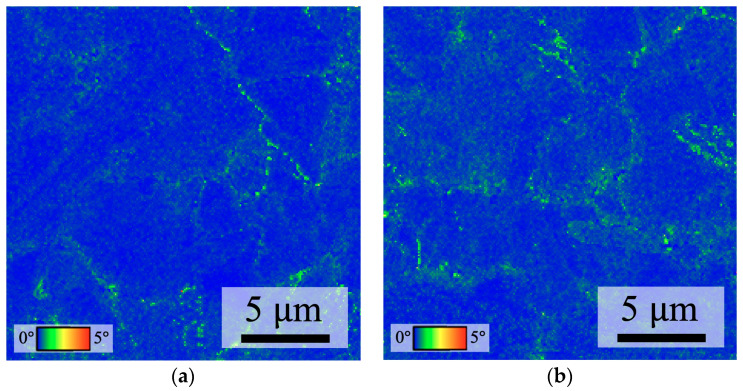
Distribution of KAM: (**a**) BCu and (**b**) QBCu.

Figure 2 illustrates the distribution of lattice misorientation, analyzed using KAM analysis, a useful EBSD mode for qualitatively estimating elastic strain based on lattice misorientation [22]. KAM is calculated as the average misorientation angle between every voxel and all neighboring points in space. For each voxel, any values exceeding the threshold angle of 5° are excluded from the averaging to suppress contributions from high-angle grain boundaries [22,23,24]. It was observed that the distribution of large KAM values was pervasive on the surface of the QBCu film, with an average value of 0.297, higher than the average value of 0.256 for the BCu film. This result also confirmed that the quenching treatment induced an increase in strain within the Cu film [25].

### 3.3. Crystallographic Orientation of Cu Films

**Figure 3:**
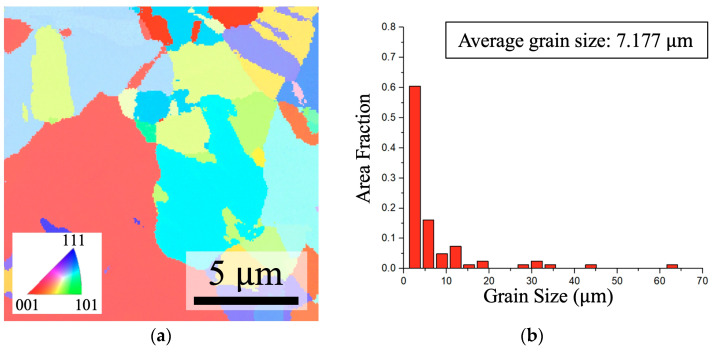

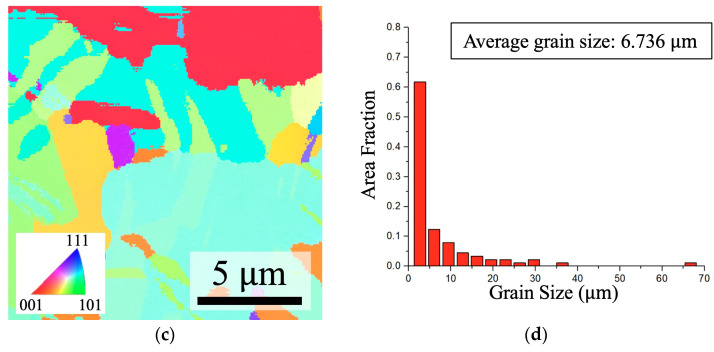
Plane views of BCu and QBCu. (**a**,**c**) Maps of the inverse pole figure from EBSD for BCu and QBCu, respectively. (**b**) Grain size distribution for the grains in (**a**). (**d**) Grain size distribution for the grains in (**c**).

SEM-EBSD analysis was conducted to characterize the crystallographic orientation and grain size of BCu and QBCu. Figure 3 shows the plane-view EBSD micrographs of both BCu and QBCu, revealing a random orientation without any particular preferred orientation. The average grain sizes of BCu and QBCu were measured to be 7.177 µm and 6.736 µm, respectively, with no significant changes observed. The grain size distribution confirmed no significant changes after the quenching treatment process, indicating that no significant recrystallization occurred.

### 3.4. Effect of Strain Energy on Bonding Interface

**Figure 4:**
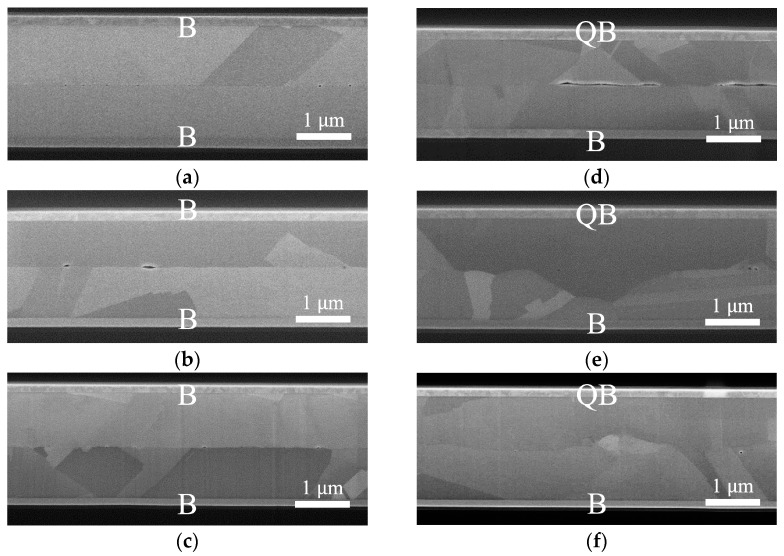
Cross-sectional SEM images: B/B bonded at 300 °C for (**a**) 1 h, (**b**) 2 h, and (**c**) 4 h. QB/B bonded at 300 °C for (**d**) 1 h, (**e**) 2 h, and (**f**) 4 h.

The cross-sectional SEM images in Figure 4 provide insight into the bonding interfaces after samples were bonded at 300 °C for 1, 2, and 4 h. Voids were found at the bonding interface, particularly in the QB/B bonded sample, as shown in Figure 4d. The presence of voids was attributed to the high roughness of the QBCu surface before bonding.

As shown in Figure 4a–c, the B/B bonded sample exhibited a clear and distinct bonding interface, maintaining a microstructure almost identical to the as-deposited BCu films. The bonding interface retained a flat plane, indicating a relatively weak bonding interface with limited diffusion between the two BCu films [16,26]. In contrast, for the bonded QB/B samples, the bonding interface exhibited a zigzag shape due to migration, attributed to grain growth and Cu interdiffusion, as illustrated in Figure 4e,f. The extent of these zigzag interfaces expanded with increasing bonding time.

**Figure 5:**
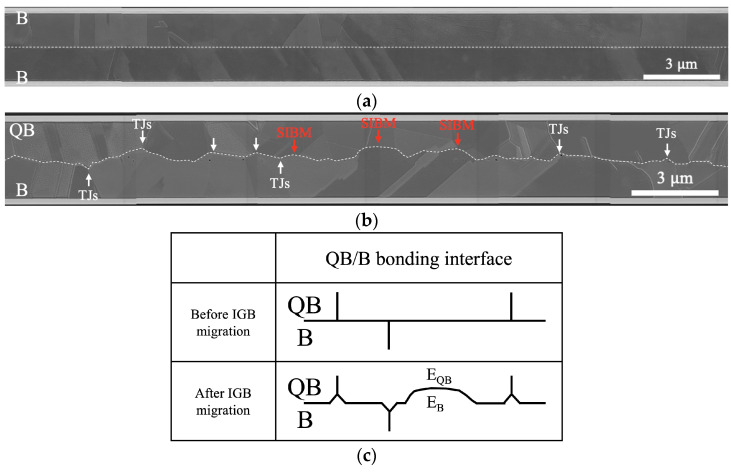
Cross-sectional SEM images of samples bonded at 300 °C for 2 h: (**a**) B/B bonded sample and (**b**) QB/B bonded sample. (**c**) Illustration of the bonding interface migration of the QB/B bonded sample. The bonding interface tended to migrate towards QBCu because atoms diffuse from regions of high strain energy to regions of low strain energy (E_QB_ > E_B_).

To confirm that the bonding interface migration through grain growth behavior was not localized, we examined cross-sectional SEM images exceeding 200 μm (partial results shown in Figure 5). The results were consistent with Figure 4b,e. Figure 5a reveals that the bonding interface of the B/B bonded sample still appears as a straight line. In contrast, the bonding interface of QB/B bonded sample has been eliminated due to migration, as shown in Figure 5b.

Bonding interface migration occurred at two sites: (1) triple junctions and (2) due to the different stored strain energies of E_QB_ and E_B_ on opposite sides of the bonding interface. As shown in Figure 5c, the differing driving forces for these two types of bonding interface migration resulted in the formation of wedge and spherical cap morphologies, respectively. The formation of wedges aimed to reduce the energy of triple junctions. The system tended to rearrange triple junctions so that the grain boundary angles were evenly distributed, typically around 120°, assuming uniform surface energy for all grain boundaries [27].

On the other hand, the formation of the spherical cap arose from strain-induced boundary migration (SIBM) [28,29]. This phenomenon was attributed to the difference in stored strain energy induced by the quenching process. Atoms tended to diffuse from regions of high strain energy to regions of low strain energy (E_QB_ > E_B_), which explained the upward bulging phenomenon of the bonding interface, as is obvious from Figure 5b, and is illustrated in the Figure 5c QB/B bonded sample.

## 4. Conclusions

A simple and effective process for fabricating three-dimensional integrated circuits, which involved the formation of wrinkles at the top of the Cu layer, has been developed through the study of Cu–Cu bonding. The occurrence of wrinkles on the QBCu surface was attributed to the differential thermal expansion between the Si substrate and the Cu film. Rapid contraction of the Cu film upon quenching from 250 °C to 0 °C induced strain along its surface, resulting in the appearance of wrinkles due to stress. This stress caused distortions in the local crystal symmetry and introduced strains and crystallographic defects in the Cu film.

Subsequent Cu bonding at 300 °C revealed grain growth at the Cu–Cu bonding interface after 2 and 4 h of bonding. The mechanism behind grain growth involved the formation of wedges, intended to reduce the energy of the triple junction and the different stored strain energies of the E_QB_ and E_B_ on opposite sides of the bonding interface.

In summary, this quenching process allows for the fabrication of a three-dimensional integrated circuit with enhanced mechanical strength of Cu joints at low temperatures.

## Data Availability

The data supporting the findings of this study are available from the corresponding author upon reasonable request.
